# Soil Water Contents Control the Responses of Dissolved Nitrogen Pools and Bacterial Communities to Freeze-Thaw in Temperate Soils

**DOI:** 10.1155/2020/6867081

**Published:** 2020-03-11

**Authors:** Nan Jiang, Yinghua Juan, Lulu Tian, Xiaodong Chen, Wentao Sun, Lijun Chen

**Affiliations:** ^1^Institute of Applied Ecology, Chinese Academy of Sciences, Shenyang, 110016 Liaoning, China; ^2^Institute of Plant Nutrition and Environmental Resources, Liaoning Academy of Agricultural Sciences, Shenyang, 110161 Liaoning, China; ^3^Department of Soil and Environment, Shenyang Agricultural University, Shenyang, 110866 Liaoning, China

## Abstract

**Background:**

Freeze-thaw influences soil-dissolved nitrogen (N) pools due to variations in bacterial communities in temperate regions. The availability of soil water is important to soil biogeochemical cycles under frozen conditions. However, it is unclear how soil water content (SWC) mediates the effects of freeze-thaw on soil-dissolved N pools and bacterial communities.

**Method:**

In this study, freeze-thaw microcosms were incubated at three levels of SWC, including 10% (air-dried soils), 15% (natural SWC), and 30% (wet soils). In addition to measuring soil-dissolved N pools, variations in bacterial communities were examined using high-throughput sequencing. *Results and Conclusions*. Total dissolved N (TDN), NO_3_^−^-N, NH_4_^+^-N, microbial biomass N (MBN), and net N mineralization rate (NNMR) were significantly influenced by SWC, freeze-thaw, and their interaction (NH_4_^+^-N excluded). N immobilization was inhibited under both low and high SWC, which was accompanied by varied bacterial community composition. However, only higher SWC substantially modified the freeze-thaw effects on the soil-dissolved N pools, characterized by a decrease in N mineralization (especially for the content of NO_3_^−^-N and NNMR) and an increase in N immobilization (MBN). These scenarios could be significantly correlated to variations in bacterial community composition based on redundancy analysis, especially by species belonging to *Bacteroidetes*, *Nitrospirae*, *Alphaproteobacteria*, *Gemmatimonadetes*, and *Verrucomicrobia* (Spearman's correlations). In conclusion, bacterial species passed through biotic (bacterial species) and abiotic filters (soil N pools) in response to freeze-thaw under varied SWC.*Bacteroidetes*, *Nitrospirae*, *Alphaproteobacteria*, *Gemmatimonadetes*, and *Verrucomicrobia* (Spearman's correlations). In conclusion, bacterial species passed through biotic (bacterial species) and abiotic filters (soil N pools) in response to freeze-thaw under varied SWC.*Nitrospirae*, *Alphaproteobacteria*, *Gemmatimonadetes*, and *Verrucomicrobia* (Spearman's correlations). In conclusion, bacterial species passed through biotic (bacterial species) and abiotic filters (soil N pools) in response to freeze-thaw under varied SWC.*Alphaproteobacteria*, *Gemmatimonadetes*, and *Verrucomicrobia* (Spearman's correlations). In conclusion, bacterial species passed through biotic (bacterial species) and abiotic filters (soil N pools) in response to freeze-thaw under varied SWC.*Gemmatimonadetes*, and *Verrucomicrobia* (Spearman's correlations). In conclusion, bacterial species passed through biotic (bacterial species) and abiotic filters (soil N pools) in response to freeze-thaw under varied SWC.*Verrucomicrobia* (Spearman's correlations). In conclusion, bacterial species passed through biotic (bacterial species) and abiotic filters (soil N pools) in response to freeze-thaw under varied SWC.

## 1. Introduction

In the temperate zone, soils are exposed to freeze-thaw (FT) during late autumn and early spring as well as during mild winters [1]. Soil freezing destroys microorganisms and releases their nutrients into soils [2]. These extra nutrients would then increase the activity of any surviving microbes while the soil thaws [3]. Accordingly, a meta-analysis suggested that FT events could significantly enhance soil-dissolved N pools and N mineralization but decrease microbial N [4]. In addition, the composition of bacterial communities could actively adjust after FT treatments, especially in temperate regions [5–7]. For example, the predominant phylum shifted from *Actinobacteria* to *Proteobacteria* and *Gemmatimonadetes* after FT treatments in our previous study [5]. Therefore, FT may influence soil N availability via changing soil bacteria, thereby influencing plant growth during subsequent growing seasons [8].

Soil water content (SWC) is important for biological reactions and nutrient transport in soils [9]. Studies suggested that SWC could influence the temperature distribution, and an increase in SWC may elevate the freezing point of soil [1, 10]. In addition, SWC could cause a direct effect on microbial growth due to its effect on the diffusion of nutrients and oxygen during soil FT events [1, 2]. There are several pieces of evidence that increasing soil moisture significantly effects FT-related N conversions in boreal soils, including NH_4_^+^-N accumulation, NO_3_^−^-N decline, and gaseous N loss [3, 11, 12]. These results are always attributed to the anaerobic conditions, which are provided by higher content of soil water and could be favorable for denitrification by microbes [3, 13]. In addition, our previous study found completely opposite changes in the bacterial communities in response to FT at two extremes of SWC [5]. Typically, the relative abundance of the active phylum *Actinobacteria* increased, decreased, and remained unchanged after FT in dry, normal, and wet soils, respectively [5]. Alternatively, as a diverse stress-tolerant bacterium, *Acidobacteria* changed in proportion contrariwise [5, 14]. Therefore, we hypothesized that SWC could influence the responses of soil N availability to FT events via changing bacterial communities. Future climate changes will likely be characterized by variable precipitation patterns, which are critical for the amount of water in soils [15]. However, there is still little information about the role of SWC in the effects of FT on soil-dissolved N pools and bacterial communities, especially in the temperate regions [4].

In this laboratory study, a temperate agricultural soil from northeast China was subjected to an FT event at three levels of soil gravity water content, accounting for 10%, 15%, and 30%. In addition to soil-dissolved N pools, we evaluated the effects of SWC on the bacterial communities in response to FT using Illumina high-throughput sequencing. The correlations between the bacterial communities and soil available N pools were further analyzed to examine the role of SWC in changing bacterial communities and soil N cycling after FT in temperate regions.

## 2. Materials and Methods

### 2.1. Soil Sampling

The study site consisted of four plots (each 20 × 10 m) under a continuous corn system in Changtu County, Liaoning Province, China (42°46′33^″^ N, 123°57′39^″^ E). The area is situated in a midtemperate, semihumid, continental monsoon climate. The area has an average frost-free period of 148 days and an average annual precipitation of 500-600 mm. The soil is classified as a Luvisol under the Food and Agriculture Organization of the United Nations [16], with 29.5 ± 0.4% field water-holding capacity, 1.23 ± 0.08 g · kg^−1^ total N, and a pH (at a 1 : 2.5 soil to water ratio) of 6.14 ± 0.03.

Soil samples from 0 to 10 cm depth were collected in late October 2013. A total of 20 soil cores in each plot were randomly collected using a 10 cm auger and were mixed into one composite sample. The samples were immediately homogenized through a 2 mm sieve to remove plant debris, large roots, and stones. Each soil sample was divided into two parts: one part (approximately 50 g) was air dried for soil analysis and the other was stored at 4°C for FT microcosm within two weeks.

### 2.2. Freeze-Thaw Microcosms

For each plot, approximately 200 g (dry weight) soil microcosms was assigned to three levels of water content: 10% (air dried in a 4°C incubator), 15% (nearly the natural SWC), and 30% (adding sterile deionized water). The values of SWC separately accounted for 34%, 50%, and 102% of field water-holding capacity. The freezing and thawing temperatures were selected based on the mean (-3.4°C) and the average maximum (1.5°C) atmospheric temperatures between October and April (2011-2012). Specifically, the soil samples were incubated at -3°C for six days and then at 2°C for another day [17]. One FT cycle was performed, providing a simulation of the periods of frozen soils driven by changing weather, especially in mild winter or early spring [1]. For controls, soil samples were constantly incubated at 2°C with the same levels of SWC. W10F, W10C, W15F, W15C, W30F, and W30C indicate the soil samples with and without freeze-thaw at a 10%, 15%, and 30% soil water content, respectively.

### 2.3. Measurement of N-Related Properties

N-related properties were measured immediately after freeze-thaw incubations. The fresh soil samples were shaken with 2 M KCl (1 : 10 *w*/*v* soil-to-solution ratio) for one hour at room temperature [18]. The filtered extracts were analyzed for NO_3_^−^-N and NH_4_^+^-N using a Continuous-Flow AutoAnalyzer (AA3, Bran & Luebbe, Norderstedt, Germany) [19]. In addition, ten milliliters of soil extract were digested using equal volumes of 73 mM alkaline potassium persulfate (containing 75 mM sodium hydroxide) in an autoclave at 121°C for 30 min and then analyzed for the dissolved total N (DTN) using a UV-2700 spectrophotometer (Shimadzu, Kyoto, Japan) [18]. The MBN was calculated from (1 day chloroform-N)/0.54 according to the chloroform-fumigation extraction method [20]. The net N mineralization rate (NNMR) of the soil was calculated as the difference in the sum of NO_3_^−^-N and NH_4_^+^-N before and after FT incubation per day [21].

### 2.4. Illumina Sequencing of 16S rRNA

Total DNA of each sample was extracted using a FastDNA Spin Kit for Soil (MP Biomedicals, Solin, OH, USA) following the manufacturer's instructions. The V4 hypervariable region of the bacterial 16S rRNA was amplified using the primers 520F and 802R (5′-AYTGGGYDTAAAGNG-3′ and 5′-TACNVGGGTATCTAATCC-3′) described before [5]. A total of 25 *μ*L of reaction was consisted of 5.0 *μ*L of 5 × PCR buffer, 2.0 *μ*L of dNTP (2.5 mM), 5.0 *μ*L of 5 × PCR GC-high enhancer, 1.0 *μ*L of each primer (10 *μ*M), 2.0 *μ*L of template genomic DNA (~200 ng/*μ*L), 0.25 *μ*L of TaKaRa polymerase (5 U/*μ*L), and 8.75 *μ*L of sterilized water. Triplicate PCR amplifications of each sample were performed with an initial denaturation at 98°C for 3 min; a total of 27 cycles of 98°C for 30 s, 50°C for 30 s, and 72°C for 30 s; and a final extension at 72°C for 5 min. Pooled triplicate reactions for each sample were purified using an AxyPrep DNA Gel Extraction Kit (Axygen Biosciences, Union City, CA, USA) and then checked for quality and quantified using a QuantiFluor™-ST fluorometer (Promega, Madison, WI, USA) and an Agilent 2100 Bioanalyzer (Agilent Technologies, Santa Clara, CA, USA). DNA libraries were constructed and evaluated using a 500-cycle (2 × 250 paired ends) kit on an Illumina MiSeq platform at a sequencing company (Personalbio Technology Co., Ltd., Shanghai, China).

### 2.5. Data Analysis

The merged raw sequences were trimmed using the Quantitative Insights Into Microbial Ecology (QIIME) toolkit v.1.7.0 to remove the sequences that were shorter than 150 bp, contained ambiguous bases, or exhibited a homopolymer longer than 8 bp [22]. Chimeric sequences were identified and removed using UCHIME in mothur (version 1.31.2, https://www.mothur.org/) [23]. The operational taxonomic units (OTUs) were clustered with a 97% similarity cutoff using the program UCLUST in QIIME [24]. The OTUs were searched against the SILVA database (Release 119) [25] for taxonomic assignment in QIIME [22].

The sequences were normalized according to the sample with the lowest number of sequences. The bacterial alpha diversity was estimated by Chao1 [26] and Shannon [27] based on the OTU data of each sample using mothur. The effects of soil water content and freeze-thaw on soil and bacterial indices were analyzed using a two-way ANOVA and PERMANOVA in SPSS 16.0 (SPSS, Chicago, IL, USA). Spearman's correlation, Duncan's test, and Kruskal-Wallis test were calculated using SPSS 16.0. To compare the beta diversity, the dissimilarities of the Bray-Curtis distance were analyzed using ANOSIM and PERMANOVA (permutations = 9999) using the vegan package in R (version 3.2.5, https://www.r-project.org). Redundancy analysis (RDA) was performed to show the correlations between the soil N properties and bacterial community composition using the vegan package in R. The partial correlation network of bacterial community composition was analyzed using the GeneNet package in R and further mapped using Cytoscape (version 3.4.0). The characteristics of the network, including number of nodes (*N*), number of links (*E*), network diameter (*d*), characteristic path length (*l*), density (*p*), clustering coefficient (*C*), and betweeness centrality (*Bi*), were calculated by NetworkAnalyzer using Cytoscape [28]. The indices *d* and *l* are the maximal path length between two nodes and the average path length over all pairs of nodes, respectively [29]. If *d* ≪ *N* − 1, the diameter of the network is small. The *p* of the network is defined as *E*/the possible links (i.e., *N*∗(*N* − 1)/2). *C* is an average of *C* (node *i*) over all nodes; *C* (node *i*) = actual number of links between neighbors of node *i*/max possible number of links between neighbors of node *i*. If *C* ≫ *p*, clustering of the network is high. Small diameter and high clustering characterize a large interconnected network [29]. Bi is calculated according to the following formula: Bi = ∑_*st*_(*σ*st(*i*)/*σ*st), where *σ*st (*i*) is the number of shortest paths from the node *s* to node *t* via node *i*, and *σ*st is the total number of shortest paths between nodes *s* and *t* [29]. Bi is always used to determine the relative importance of nodes [29].

## 3. Results

### 3.1. Soil N Properties in Response to Freeze-Thaw at Different Contents of Soil Water

**Figure 1:**
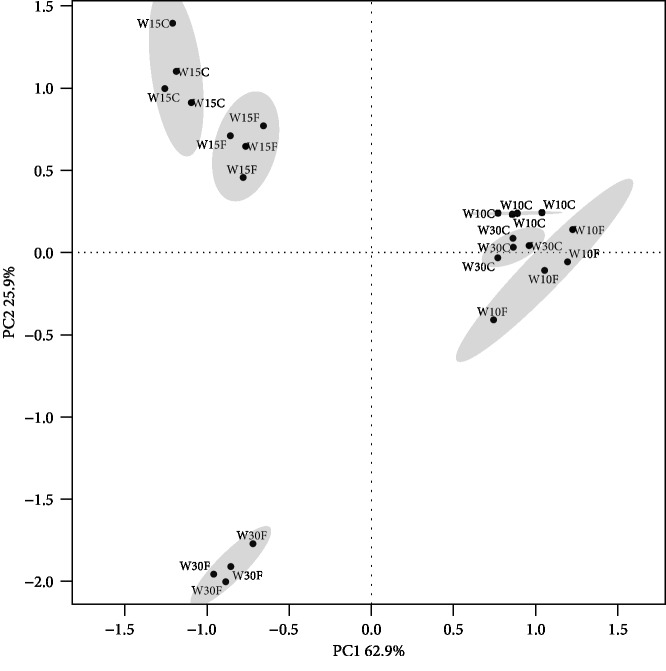
Principal coordinate analysis (PCoA) plot of the soil properties in each soil sample. Ellipses have been drawn for each treatment with a confidence limit of 0.95.

SWC, FT, and the interaction of both SWC and FT significantly influenced NNMR, the contents of NO_3_^−^, DTN, and MBN (Table 1). The NH_4_^+^ content was only significantly affected by SWC and FT (Table 1). In addition, the PCoA plot indicated that all the examined indices could be well separated by both SWC and FT (Figure 1). In particular, the examined indices were most affected by FT at SWC of 30% (Figure 1). PERMANOVA indicated that SWC, FT, and their interaction attributed to 71.9%, 10.4%, and 14.4% of the variations in the examined N indices, respectively (*P* < 0.001).

### 3.2. Alpha and Beta Diversity of Soil Bacterial Communities in Response to FT at Different SWCs

**Figure 2:**
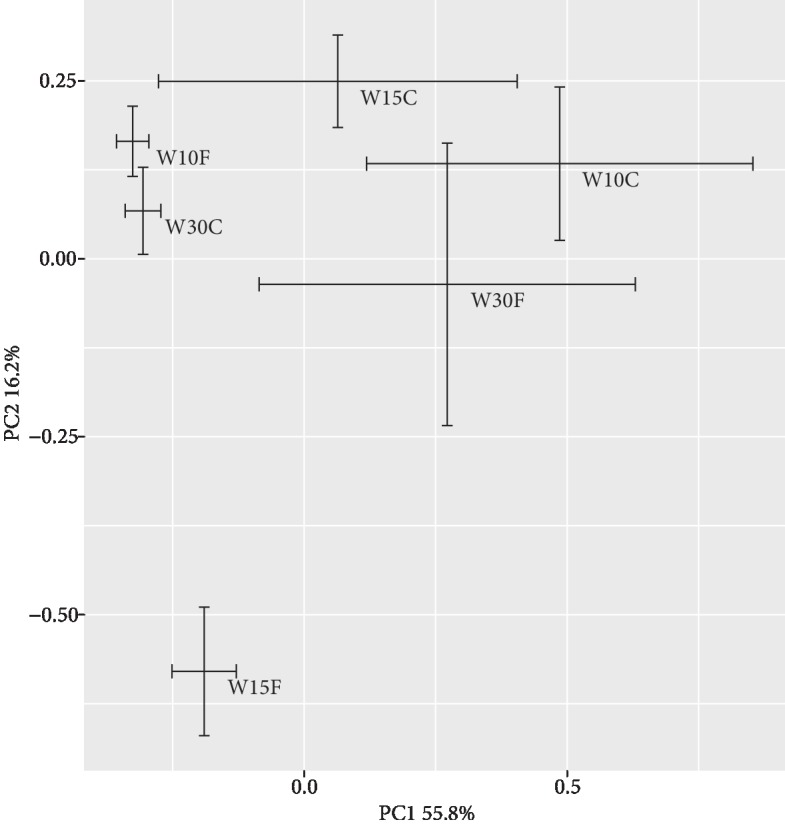
Principal coordinate analysis (PCoA) plot of bacterial community composition in each soil sample. The position of each point is the mean of four replicates. Error bars indicate the standard error.

Following the parallel sequencing of 24 soil samples, a total of 1,645,533 trimmed sequences were observed (2043-3542 OTUs per sample). The bacterial richness estimated by Chao1 ranged from 2750 in the W30F to 3193 in the W10F. Two-way ANOVA suggested that Chao1 was only significantly influenced by SWC (*F* = 4.2, *P* < 0.05), with a negative correlation (Spearman's correlation: -0.52, *P* < 0.05). There was no significant effect of SWC (*P* = 0.58), FT (*P* = 0.36), or the interaction of both SWC and FT (*P* = 0.27) on the bacterial diversity estimated by Shannon (two-way ANOVA). The bacterial beta diversity based on the Bray-Curtis distance was significantly different between treatments (ANOSIM R: 0.45, *P* < 0.001) (Figure 2). PERMANOVA further indicated that SWC, FT, and the interaction between SWC and FT accounted for 19.0% (*F* = 3.0, *P* < 0.001), 7.7% (*F* = 2.5, *P* < 0.05), and 17.1% (*F* = 2.7, *P* < 0.01) of the variations in the bacterial beta diversity, respectively.

### 3.3. Bacterial Community Composition in Response to FT at Different SWCs

**Figure 3:**
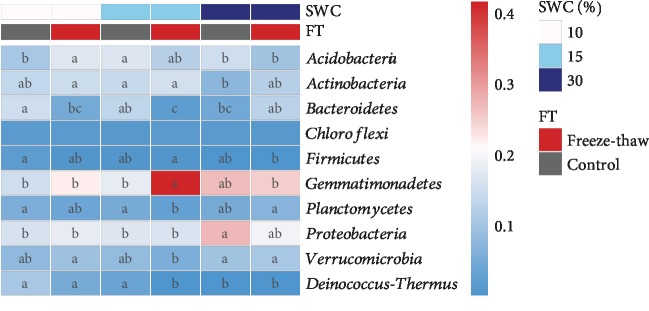
The relative abundance of dominant bacterial phyla (averagely >1%) in each treatment. The letters within each row indicate the significant differences among all treatments (Kruskal-Wallis test, *P* < 0.05).

The majority of sequences (96.2–98.4% in all) were affiliated into 10 main phyla (relative abundance > 1% averagely, Figure 3): *Gemmatimonadetes* (15.0–41.4%), *Proteobacteria* (16.1–27.3%), *Acidobacteria* (9.9–17.0%), *Actinobacteria* (7.4–15.4%), *Bacteroidetes* (1.9–15.0%), *Verrucomicrobia* (5.7–10.7%), *Planctomycetes* (2.5–6.8%), *Deinococcus-Thermus* (0.02–10.5%), *Chloroflexi* (1.0–1.3%), and *Firmicutes* (0.3–1.8%). The interaction between SWC and FT had a significant effect on the relative abundance of four phyla, including *Acidobacteria* (*F* = 4.12, *P* < 0.01), *Bacteroidetes* (*F* = 5.65, *P* < 0.05), *Gemmatimonadetes* (*F* = 3.84, *P* < 0.05), and *Deinococcus-Thermus* (*F* = 5.15, *P* < 0.01). Specifically, the relative abundance of *Acidobacteria* significantly increased in response to FT at SWC of 10%, while that of *Deinococcus-Thermus* and *Gemmatimonadetes* decreased and increased, respectively, in response to freeze-thaw treatment at SWC of 15% (*P* < 0.05). Alternatively, the relative abundance of *Bacteroidetes* decreased in response to FT at 10% and 15% SWCs but increased at SWC of 30%. In addition, SWC significantly influenced the relative abundance of both *Actinobacteria* (*F* = 6.21, *P* < 0.01) and *Firmicutes* (*F* = 3.08, *P* < 0.05).

Among the 171 main OTUs (of relative abundance > 0.1% averagely), a total of 120 OTUs were significantly influenced by SWC, FT, or their interaction ([Supplementary-material supplementary-material-1]). Specifically, eight OTUs, affiliated into four phyla *Gemmatimonadetes*, *Bacteroidetes*, *Actinobacteria*, and *Proteobacteria*, were significantly influenced by SWC, FT, and the interaction of both SWC and FT. Another 74 OTUs, belonging to the phyla *Verrucomicrobia*, *Proteobacteria*, *Gemmatimonadetes*, *Firmicutes*, *Bacteroidetes*, *Actinobacteria*, and *Acidobacteria*, were significantly influenced by the interaction between SWC and FT ([Supplementary-material supplementary-material-1]).

### 3.4. Correlations between Soil Properties and Bacterial Communities

**Figure 4:**
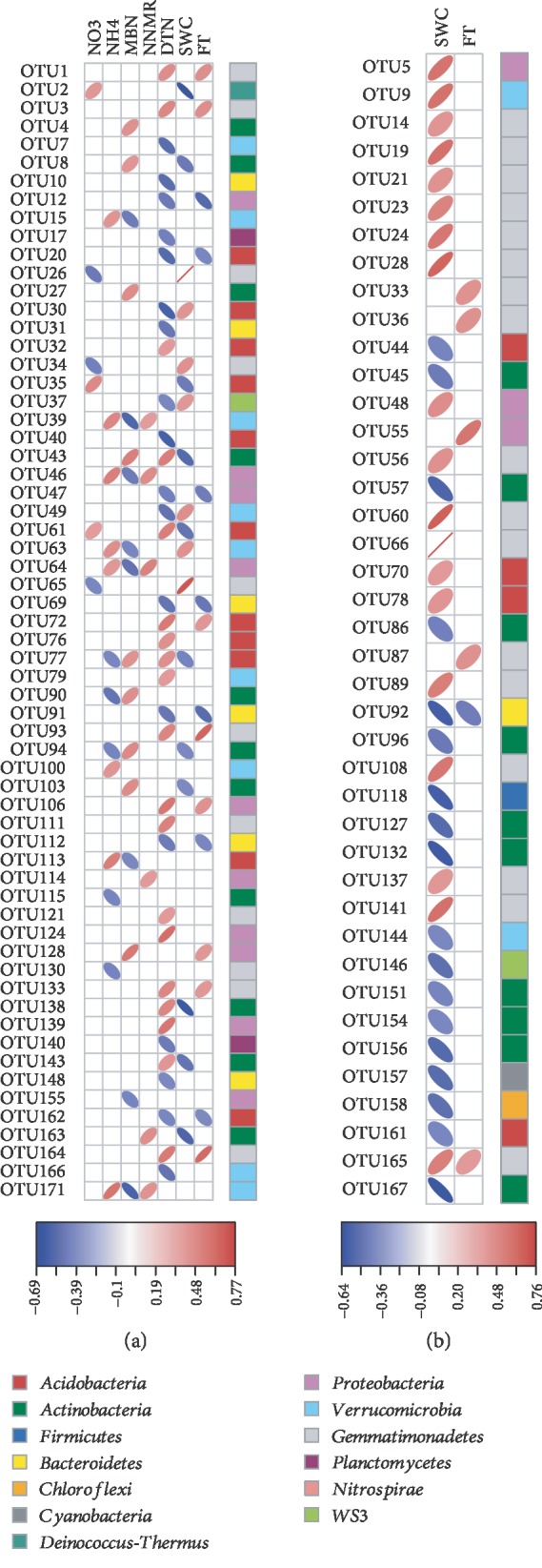
Spearman's correlations between OTUs and soil properties. Only significant correlations to (a) at least one N index or to (b) one or both of the soil water content (SWC) and freeze-thaw treatment (FT) are shown (*P* < 0.05). MBN: microbial biomass N; NNMR: net N mineralization rate; DTN: dissolved total N; SWC: soil water content; FT: freeze-thaw treatment.

Bacterial alpha-diversity index Chao1 showed a significant Spearman's correlation to the content of NO_3_^−^ (*r* = 0.45, *P* < 0.05). PERMANOVA suggested that DTN was significantly related to bacterial beta diversity (*P* < 0.05). According to Spearman's correlations, a total of 62 OTUs were significantly related to at least one of the N indices (Figure 4(a)). In addition, another 37 and six OTUs were significantly related to SWC and FT, respectively (Figure 4(b)). RDA analyses suggested that all of the examined soil N properties significantly retained the variations in the bacterial community composition (Table 2). In addition, SWC showed a strong and significant effect on the bacterial community composition (*r*^2^ = 0.72, *P* < 0.001). FT had a significant but weak effect on the bacterial community composition (*r*^2^ = 0.16, *P* < 0.05).

In the partial correlation network, a total of 333 significant correlations were detected with regard to 142 OTUs ([Supplementary-material supplementary-material-1]). The network parameters are shown in Table 3. The clustering coefficient (*C*) was 0.363, far greater than *p* and suggesting a high clustering. The network diameter (*d*) was lower than 148 (N-1). OTUs were always clustered with members belonging to the same phylum ([Supplementary-material supplementary-material-1]). In particular, OTU100 and OTU128 (belonging separately to the phyla *Verrucomicrobia* and *Proteobacteria*), followed by OTU139 and OTU162 (belonging separately to the phyla *Proteobacteria* and *Acidobacteria*) harbored higher values of Bi than other nodes. In addition, FT, DTN, and NO_3_^−^ also had relatively high values of Bi.

## 4. Discussion

### 4.1. Varied SWC Reduced Microbial N Assimilation and Changed the Bacterial Communities

Soil water is a fundamental and important parameter of soil biogeochemistry under frozen conditions [9]. In this study, SWC significantly influenced dissolved N pools. The N mineralization rate and inorganic N pools such as NO_3_^−^-N and NH_4_^+^-N were always lowest in soils with natural SWC (i.e., 15%). The microbial biomass of N in soils with 15% SWC was approximately double the value that was in soils with either higher or lower SWC. Microbial growth and development are limited when substrate diffusion is limited by low availability of soil water; however, they can also be limited when high soil water content restricts the diffusion of oxygen [30]. Specifically, in drier soils (i.e., 10% in this study), bacteria have to survive from the shortage of nutrients. However, in wetter soils (i.e., 30% in this study), they have to adapt quickly to the oxygen-deficient environment. Therefore, a proper value of SWC would partially maintain the assimilation of soil N by indigenous microbes at low temperatures.

Sudden changes in SWC can induce stress on bacteria, which favors the growth of certain taxa, and drastically reduces microbial N [15]. In particular, bacterial alpha-diversity index Chao1 representing OTU richness was significantly lower in soils at 30% SWC in this study. In addition, the proportion of six phyla, including *Actinobacteria*, *Firmicutes*, *Deinococcus-Thermus*, *Acidobacteria*, *Bacteroidetes*, and *Gemmatimonadetes*, was significantly influenced by the varied content of soil water. The changes of bacterial communities with varied SWCs provided the premise to test our hypothesis that bacteria is one key in the responses of available N to FT. In particular, a total of 29 OTUs (belonging to *Acidobacteria*, *Actinobacteria*, *Deinococcus-Thermus*, *Bacteroidetes*, *Chloroflexi*, *Firmicutes*, *Verrucomicrobia*, *Cyanobacteria*, and WS3) had positive responses to decreasing contents of soil water. In addition, a total of 26 OTUs (belonging to *Acidobacteria*, *Gemmatimonadetes*, *Proteobacteria*, *Verrucomicrobia*, and SW3) had positive responses to increasing SWCs. *Actinobacteria*, *Firmicutes*, and *Deinococcus-Thermus* had been reported to be resistant to desiccation [31–33], while *Acidobacteria*, *Alphaproteobacteria* and *Gammaproteobacteria*, and *Verrucomicrobia* always followed an opposite pattern [14, 31, 34]. This is reflected in the majority of our results. However, five acidobacterial OTUs still showed negative responses to increasing SWCs in this study. *Gemmatimonadetes* had been suggested to prefer dryer soils [35], but opposite trends were still detected in this and some other studies [5, 31]. Disparate responses among members were also observed in *Verrucomicrobia* and WS3 in the study. Species of these phyla are equipped with fermentative and respiratory abilities [36, 37]. These results suggest that certain bacteria, especially those with facultative anaerobic capabilities, are active in response to, but not strictly limited by, varied contents of soil water.

### 4.2. Higher Content of Soil Water Substantially Modified the Effects of FT on Soil-Dissolved N Pools due to Variations in Bacterial Community Composition

Similar to most previous studies, FT caused significant increases in NH_4_^+^ from soils with all content of SWC. Likewise, the contents of dissolved total N also significantly increased from soils with low and natural SWCs. The scenarios that were attributed to were that FT could destroy bacterial cells and increase mineralization and the risk of N leaching [4]. Furthermore, our results suggested that the lower SWC only narrowed the positive effects of FT on soil-dissolved N pools. However, the highest value of SWC (i.e., 30% in this study) substantially modified the responses of N indices to FT. For instance, the N mineralization rate increased by 17.1% at the natural SWC but decreased by 37.6% at a higher content of soil water after one FT cycle. Alternatively, neither NO_3_^−^ nor MBN significantly changed after FT at the natural SWC, while NO_3_^−^ content fell 26.3% and MBN increased 86.7% after FT at a higher content of soil water. As discussed above, high content of soil water results in oxygen deficiency that may limit the activity of aerobic bacteria and simultaneously favor denitrification [4, 8, 12]. This was consistent with the opposite responses of the mineralization rate to FT at natural and high SWCs. Approximately half of the OTUs were significantly influenced by the interaction between SWC and FT. For example, the relative abundance of OTU59, belonging to the nitrifying bacterial phylum *Nitrospirae* [38], increased after FT in soils with SWC of 10% and 15%. However, the relative abundance was lowest in soils at 30% SWC, without any effects from FT. Similar results were also detected in the relative abundance of several OTUs assigned into the genus *Phenylobacterium* (*Alphaproteobacteria*), which are bacteria that use ammonium and nitrate directly [39]. In contrast, several OTUs belonging to the phylum *Bacteroidetes* (esp. in the family *Chitinophagaceae*), many members of which are devoted to denitrification [40], decreased after FT in soils with natural content of soil water. The relative abundance was unchanged or even increased in drier soils. The two scenarios were similar to several OTUs belonging to *Gemmatimonadetes* and *Verrucomicrobia*. Relatively little information is available on either phylum since they are uncultivable [41]. Current studies speculate that the two phyla may contribute to the N cycle in freshwater or agroecosystems [42, 43]. Species (OTUs) detected here should be paid more attention to in future FT studies. Here, SWC, especially an increasing SWC, has been suggested to modify the responses of soil-dissolved N pools as well as the bacterial communities to FT, initially proving our hypothesis.

Previous studies indicated that increases in SWC decreased soil temperature at constant air temperatures above zero but increased soil temperature at constant air temperatures below zero [10]. At similar temperatures, soils with a higher content of soil water had higher freezing points and larger amounts of water movement; therefore, soils with higher content of soil water would be prone to freeze but hard to thaw [10, 44]. That is, under parallel FT microcosms, higher content of soil water may limit the amplitude of FT and increase freezing duration. Either condition has been reported to constrain NO_3_^−^-N production [4, 17, 45]. Longer frost duration may provide more sufficient time for facultative bacteria to switch from aerobic to anaerobic activities [2]. Both reducing the production and accelerating the consumption attributed to the negative response of NO_3_^−^-N content and net N mineralization rate to FT treatment in soils with a higher content of soil water in this study. Generally, the inhibition of nitrification should benefit the NH_4_^+^-N pool. A recent meta-analysis suggested that the FT effect significantly promoted the NH_4_^+^-N pools due to more mineralizable organics provided by dead cells [4]. As a result of the higher content of soil water, the increase in the NH_4_^+^-N content shrank in response to FT. In this study, the content of NH_4_^+^-N increased by 53.4% in soil with the natural SWC but only increased by 9.7% in soil with a higher SWC. This may again be attributed to the anoxic microsites caused by the higher content of soil water. In addition, some bacteria could directly uptake ammonia, amino acids, and peptides provided by dead cells as well as N mineralization through different transport systems [8, 17, 46]. Therefore, contrary to drought, higher SWC could stimulate microbial immobilization of N during the freeze-thaw process.

### 4.3. Responses of Bacterial Community Composition to FT with Varied SWC

The partial correlation network between OTUs and N indices was complicated, characterized by the higher clustering coefficient [47]. In addition, the small network diameter and characteristic path length also suggested more interdependence [29] between bacterial species (OTUs). The majority of bacterial species (OTUs) clustered with other species from the same phylum. The clustering between species within *Bacteroidetes*, *Planctomycetes*, or *Verrucomicrobia* was also detected in another study [5], which suggests that these bacteria may cooperate with their kin in response to the joint effects of SWC and FT. However, a few species assigned to *Actinobacteria*, *Acidobacteria*, *Proteobacteria*, *Chloroflexi*, and *Gemmatimonadetes* were scattered over the network and were significantly correlated to species belonging to other phyla or environmental factors. Take OTU100 and OTU128 as examples, both harbored the highest Bi in the network, indicating their important roles in the network. The former belongs to *Verrucomicrobia* and showed significant correlations to 11 OTUs belonging to five phyla. The latter belonged to *Proteobacteria* and showed significant connections to 11 OTUs belonging to seven phyla as well as DTN and MBN. This phenomenon was also observed for OTU139 and OTU162 with relatively large Bi in the network, which belong to *Proteobacteria* (the genus *Ochrobactrum*) and *Acidobacteria* (the genus *Candidatus Koribacter*), respectively. Members of these phyla were always reported to be active in frozen soils or during soil thawing [46]. These species may pass through both biotic (other species) and abiotic (environmental factors) filters as the ties holding the bacterial community stable in response to the joint effects of soil water content and freeze-thaw. Soil available N was adjusted during the processes, confirming the hypothesis that SWCs modify the responses of soil N availability to FT via changed bacterial members such as OTU100 and OTU128.

## 5. Conclusions

In this study, soil water content, freeze-thaw, and their interaction influenced both dissolved N pools and bacterial communities in temperate soils. In particular, higher soil water content substantially changed soil N transformation in response to freeze-thaw and mainly contributed to variations in the bacterial species (OTUs) devoting to either nitrification or denitrification. Regrettably, however, we conducted the freeze-thaw microcosms without a time scale, and we could not find the dynamics of species during the thawing process. In addition, the important bacteria passing through both biotic and abiotic filters were uncovered in response to freeze-thaw under different soil water contents. Whether these species are common in different geographical locations deserves further investigations.

## Figures and Tables

**Table 1:** Soil N pools (mg N kg^−1^ dry soil) and the net N mineralization rate (NNMR, mg N kg^−1^ day^−1^) for each freeze-thaw (FT) treatment (mean (standard deviation)) and results from two-way ANOVA among soil water content (SWC), FT, and their interactions (SWC × FT).

	NO_3_^−^	NH_4_^+^	DTN	MBN	NNMR
W10C	28.74 (0.19)a	7.21 (0.15)b	45.97 (0.13)bc	3.12 (0.66)c	2.60 (0.02)b
W10F	28.78 (1.01)a	8.49 (0.41)a	46.65 (0.44)ab	3.22 (0.30)c	2.78 (0.19)a
W15C	24.79 (0.30)b	2.77 (0.43)d	43.32 (1.09)e	7.82 (0.40)a	1.40 (0.07)d
W15F	25.03 (0.33)b	4.25 (0.50)c	47.29 (0.43)a	7.37 (0.31)a	1.64 (0.08)c
W30C	28.39 (0.39)a	7.43 (0.25)b	44.78 (1.05)d	2.71 (0.13)c	2.58 (0.09)b
W30F	20.91 (0.41)c	8.15 (0.04)a	45.04 (0.61)cd	5.06 (0.31)b	1.61 (0.05)c
Two-way	ANOVA				
SWC	162.3^∗∗^	436.1^∗∗^	8.2^∗^	302.4^∗∗^	290.9^∗∗^
FT	132.4^∗∗^	70.4^∗∗^	31.6^∗∗^	17.8^∗∗^	20.1^∗∗^
SWC × FT	148.2^∗∗^	2.8	16.3^∗∗^	29.3^∗∗^	99.2^∗∗^

Letters indicate the significant difference in each index among the FT treatments (Duncan's HSD test, *n* = 4, *P* < 0.05). Asterisks (^∗∗^*P* < 0.001 and ^∗^*P* < 0.01) indicate the significant influence of FT, SWC, and their interactions in two-way ANOVA.

**Table 2:** Correlations (squared correlation coefficient retrieved from the redundancy analysis) between bacterial community composition and soil N properties.

	NO_3_^−^	NH_4_^+^	DTN	MBN	NNMR
Community composition	0.66^∗∗∗^	0.32^∗^	0.50^∗∗∗^	0.55^∗∗∗^	0.30^∗^

Asterisks (^∗∗∗^*P* < 0.001 and ^∗^*P* < 0.01) indicate the significant influence.

**Table 3:** Parameters of partial correlation networks.

Number of links (*E*)	Number of nodes (*N*)	Characteristic path length (*l*)	Network diameter (*d*)	Density (*p*)	Clustering coefficient (*C*)
333	149	4.159	4.47	0.03	0.363

## Data Availability

The data used to support the findings of this study are available from the corresponding author upon request.

## Supplementary Materials

Supplementary Materials Supplementary figures include the comparisons of bacterial OTUs between different treatments and the correlations between bacterial OTUs and soil-dissolved N pools using heat maps and partial correlation network, respectively.

## References

[1] Henry H. A. L. (2007). Soil freeze-thaw cycle experiments: trends, methodological weaknesses and suggested improvements. *Soil Biology & Biochemistry*.

[2] Risk N., Snider D., Wagner-Riddle C. (2013). Mechanisms leading to enhanced soil nitrous oxide fluxes induced by freeze-thaw cycles. *Canadian Journal of Soil Science*.

[3] Koponen H. T., Martikainen P. J. (2004). Soil water content and freezing temperature affect freeze-thaw related N_2_O production in organic soil. *Nutrient Cycling in Agroecosystems*.

[4] Song Y., Zou Y. C., Wang G. P., Yu X. F. (2017). Altered soil carbon and nitrogen cycles due to the freeze-thaw effect: a meta- analysis. *Soil Biology & Biochemistry*.

[5] Juan Y. H., Jiang N., Tian L. L., Chen X. D., Sun W. T., Chen L. J. (2018). Effect of freeze-thaw on a midtemperate soil bacterial community and the correlation network of its members. *Biomed Research International*.

[6] Sharma S., Szele Z., Schilling R., Munch J. C., Schloter M. (2006). Influence of freeze-thaw stress on the structure and function of microbial communities and denitrifying populations in soil. *Applied and Environmental Microbiology*.

[7] Stres B., Philippot L., Faganeli J., Tiedje J. M. (2010). Frequent freeze-thaw cycles yield diminished yet resistant and responsive microbial communities in two temperate soils: a laboratory experiment. *FEMS Microbiology Ecology*.

[8] Urakawa R., Shibata H., Kuroiwa M. (2014). Effects of freeze-thaw cycles resulting from winter climate change on soil nitrogen cycling in ten temperate forest ecosystems throughout the Japanese archipelago. *Soil Biology and Biochemistry*.

[9] Oquist M. G., Sparrman T., Klemedtsson L. (2009). Water availability controls microbial temperature responses in frozen soil CO_2_ production. *Global Change Biology*.

[10] Chen S., Ouyang W., Hao F., Zhao X. (2013). Combined impacts of freeze-thaw processes on paddy land and dry land in Northeast China. *Science of the Total Environment*.

[11] Szukics U., Abell G. C. J., Hodl V. (2010). Nitrifiers and denitrifiers respond rapidly to changed moisture and increasing temperature in a pristine forest soil. *FEMS Microbiology Ecology*.

[12] Wu X., Bruggemann N., Butterbach-Bahl K., Fu B. J., Liu G. H. (2014). Snow cover and soil moisture controls of freeze-thaw-related soil gas fluxes from a typical semi-arid grassland soil: a laboratory experiment. *Biology and Fertility of Soils*.

[13] Li W. B., Wu J. B., Bai E. (2016). Response of terrestrial nitrogen dynamics to snow cover change: a meta- analysis of experimental manipulation. *Soil Biology & Biochemistry*.

[14] Mannisto M. K., Kurhela E., Tiirola M., Haggblom M. M. (2013). Acidobacteria dominate the active bacterial communities of Arctic tundra with widely divergent winter-time snow accumulation and soil temperatures. *FEMS Microbiology Ecology*.

[15] Evans S. E., Wallenstein M. D. (2014). Climate change alters ecological strategies of soil bacteria. *Ecology Letters*.

[16] FAO-UNESCO (1957). Soil map of the world, 1:5 000 000. *Nature*.

[17] Jiang N., Juan Y. H., Tian L. L., Chen X. D., Sun W. T., Chen L. J. (2018). Modification of the composition of dissolved nitrogen forms, nitrogen transformation processes, and diversity of bacterial communities by freeze- thaw events in temperate soils. *Pedobiologia*.

[18] Lu R. K. (2000). *Chemical Analysis Method of Agricultural Soil*.

[19] Mulvaney R. L., Sparks D. L. (1996). Nitrogen: inorganic forms. *Methods of Soil Analysis, Part 3, Chemical Methods*.

[20] Brookes P. C., Landman A., Pruden G., Jenkinson D. S. (1985). Chloroform fumigation and the release of soil nitrogen: a rapid direct extraction method to measure microbial biomass nitrogen in soil. *Soil Biology & Biochemistry*.

[21] Zhang X. L., Wang Q. B., Li L. H., Han X. G. (2008). Seasonal variations in nitrogen mineralization under three land use types in a grassland landscape. *Acta Oecologica*.

[22] Caporaso J. G., Kuczynski J., Stombaugh J. (2010). QIIME allows analysis of high-throughput community sequencing data. *Nature Methods*.

[23] Edgar R. C., Haas B. J., Clemente J. C., Quince C., Knight R. (2011). UCHIME improves sensitivity and speed of chimera detection. *Bioinformatics*.

[24] Edgar R. C. (2010). Search and clustering orders of magnitude faster than BLAST. *Bioinformatics*.

[25] Quast C., Pruesse E., Yilmaz P. (2013). The SILVA ribosomal RNA gene database project: improved data processing and web-based tools. *Nucleic Acids Research*.

[26] Chao A., Lee S. M., Jeng S. L. (1992). Estimating population-size for capture recapture data when capture probabilities vary by time and individual animal. *Biometrics*.

[27] Shannon C. E. (1997). The mathematical theory of communication. *Physics Today*.

[28] Shannon P., Markiel A., Ozier O. (2003). Cytoscape: a software environment for integrated models of biomolecular interaction networks. *Genome Research*.

[29] Pugacheva E. (2015). Social network modelling. *Interdisciplinary Studies of Complex Systems*.

[30] Lellei-Kovacs E., Botta-Dukat Z., de Dato G. (2016). Temperature dependence of soil respiration modulated by thresholds in soil water availability across European shrubland ecosystems. *Ecosystems*.

[31] Barnard R. L., Osborne C. A., Firestone M. K. (2013). Responses of soil bacterial and fungal communities to extreme desiccation and rewetting. *ISME Journal*.

[32] Mohammadipanah F., Wink J. (2016). *Actinobacteria* from arid and desert habitats: diversity and biological activity. *Frontiers in Microbiology*.

[33] Tian B., Hua Y. J. (2010). Carotenoid biosynthesis in extremophilic *Deinococcus-Thermus* bacteria. *Trends in Microbiology*.

[34] Karaoz U., Couradeau E., da Rocha U. N. (2018). Large blooms of *Bacillales*(Firmicutes) underlie the response to wetting of cyanobacterial biocrusts at various stages of maturity. *Mbio*.

[35] DeBruyn J. M., Nixon L. T., Fawaz M. N., Johnson A. M., Radosevich M. (2011). Global biogeography and quantitative seasonal dynamics of Gemmatimonadetes in soil. *Applied and Environmental Microbiology*.

[36] Farag I. F., Youssef N. H., Elshahed M. S. (2017). Global distribution patterns and pangenomic diversity of the candidate phylum "*Latescibacteria*" (WS3). *Applied and Environmental Microbiology*.

[37] Freitas S., Hatosy S., Fuhrman J. A. (2012). Global distribution and diversity of marine *Verrucomicrobia*. *ISME Journal*.

[38] Lucker S., Wagner M., Maixner F. (2010). A Nitrospira metagenome illuminates the physiology and evolution of globally important nitrite-oxidizing bacteria. *Proceedings of the National Academy of Sciences of the United States of America*.

[39] Eberspächer J., Whitman W. B. (2015). Phenylobacterium. *Bergey's Manual of Systematics of Archaea and Bacteria*.

[40] Kraft B., Tegetmeyer H. E., Meier D., Geelhoed J. S., Strous M. (2014). Rapid succession of uncultured marine bacterial and archaeal populations in a denitrifying continuous culture. *Environmental Microbiology*.

[41] Bergmann G. T., Bates S. T., Eilers K. G. (2011). The under-recognized dominance of *Verrucomicrobia* in soil bacterial communities. *Soil Biology & Biochemistry*.

[42] Chiang E., Schmidt M. L., Berry M. A. (2018). *Verrucomicrobia* are prevalent in north-temperate freshwater lakes and display class-level preferences between lake habitats. *PLoS One*.

[43] Zhou X. G., Wang Z. L., Jia H. T., Li L., Wu F. Z. (2018). Continuously monocropped *Jerusalem Artichoke* changed soil bacterial community composition and ammonia-oxidizing and denitrifying bacteria abundances. *Frontiers in Microbiology*.

[44] Nagare R. M., Schincariol R. A., Quinton W. L., Hayashi M. (2012). Effects of freezing on soil temperature, freezing front propagation and moisture redistribution in peat: laboratory investigations. *Hydrology and Earth System Sciences*.

[45] Smith J., Wagner-Riddle C., Dunfield K. (2010). Season and management related changes in the diversity of nitrifying and denitrifying bacteria over winter and spring. *Applied Soil Ecology*.

[46] Nikrad M. P., Kerkhof L. J., Haggblom M. M. (2016). The subzero microbiome: microbial activity in frozen and thawing soils. *FEMS Microbiology Ecology*.

[47] Rasskin-Gutman D., Esteve-Altava B. (2014). Connecting the dots: anatomical network analysis in morphological EvoDevo. *Biological Theory*.

