# Supplementary material for: Soil Water Contents Control the Responses of Dissolved Nitrogen Pools and Bacterial Communities to Freeze-Thaw in Temperate Soils
Source: Biomed Res Int. 2020 Mar 11;2020:6867081. doi: 10.1155/2020/6867081 (PMC7086428; doi:10.1155/2020/6867081)
Supplement: Supplementary Materials — Supplementary figures include the comparisons of bacterial OTUs between different treatments and the correlations between bacterial OTUs and soil-dissolved N pools using heat maps and partial correlation network, respectively. [file 6867081.f1.docx]

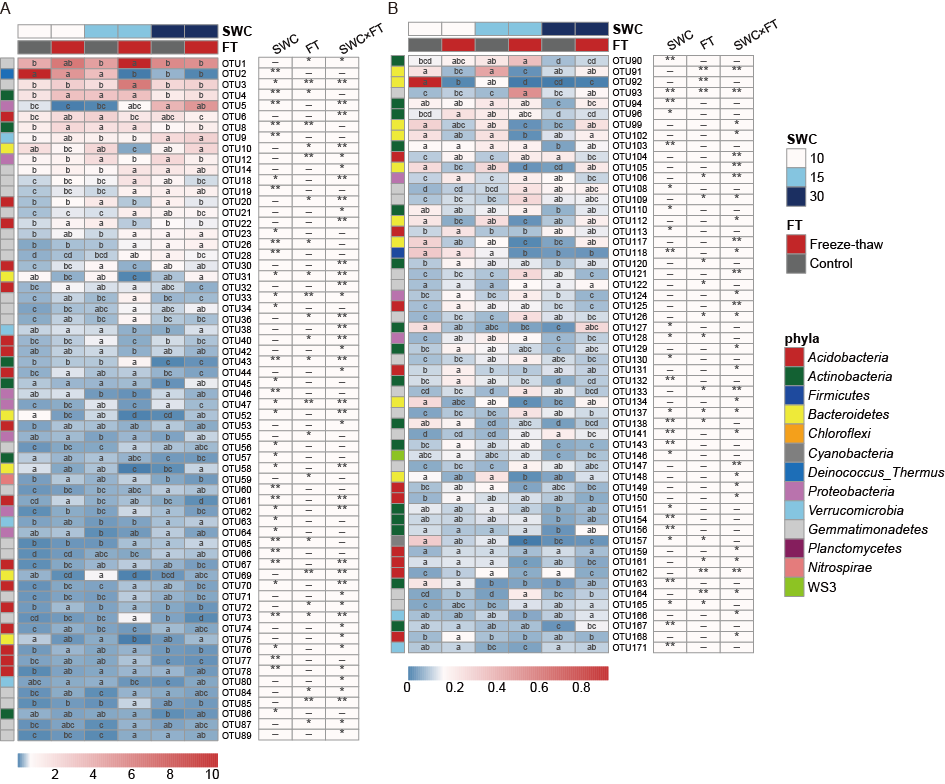
Fig. S1 Distribution of bacterial OTUs (A, of relative abundance > 0.2%; B, 0.1% < relative abundance < 0.2%) with significant correlations to soil water content (SWC), freeze-thaw (FT) or their interactions (SWC×FT) in each treatment. The right plots indicate the results of PERMANOVA. The letters within each row indicate the significant differences among all treatments (Kruskal-Wallis test, *P* < 0.05).


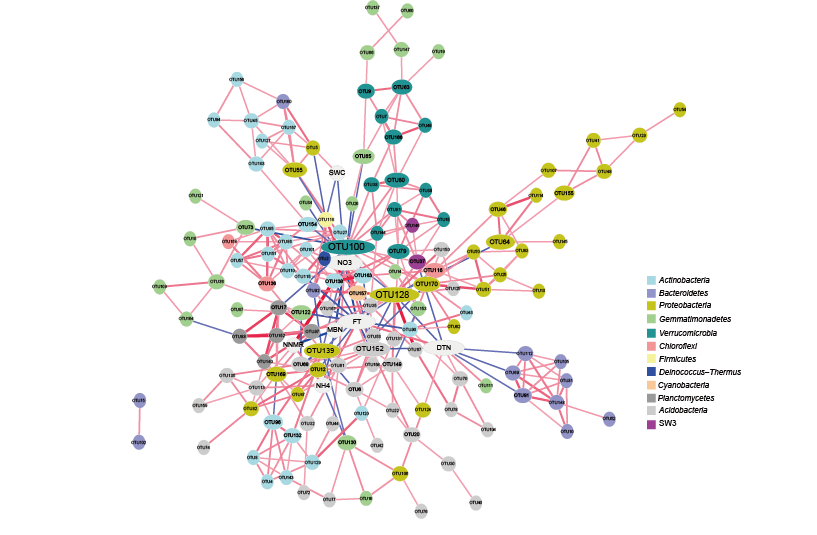
Fig. S2 Partial correlation network between soil properties and OTUs. The size of the node indicates the importance in the network based on the betweenness centrality (B*i*): the higher value of B*i*, the bigger size of the node. Different colors of nodes indicate different phyla. Positive and negative correlations are shown by red and blue lines, respectively. MBN: microbial biomass N; NNMR: net N mineralization rate; DTN: dissolved total N; SWC: soil water content; FT: freeze-thaw treatment.
